# Rare variants confer shared susceptibility to gastrointestinal tract cancer risk

**DOI:** 10.3389/fonc.2023.1161639

**Published:** 2023-07-06

**Authors:** Ji Zheng, Xin Wang, Jingrao Li, Yuanna Wu, Jiang Chang, Junyi Xin, Meilin Wang, Tianpei Wang, Qingyi Wei, Mengyun Wang, Ruoxin Zhang

**Affiliations:** ^1^ Department of Epidemiology, School of Public Health, Key Laboratory of Public Health Safety, Ministry of Education, Fudan University, Shanghai, China; ^2^ Office of Cancer Screening, National Cancer Center/National Clinical Research Center for Cancer/Cancer Hospital, Chinese Academy of Medical Sciences and Peking Union Medical College, Beijing, China; ^3^ Department of Biological Sciences, Dedman College of Humanities and Sciences, Southern Methodist University, Dallas, TX, United States; ^4^ Department of Health Toxicology, Key Laboratory for Environment and Health, School of Public Health, Tongji Medical College, Huazhong University of Science and Technology, Wuhan, Hubei, China; ^5^ Department of Environmental Genomics, Jiangsu Key Laboratory of Cancer Biomarkers, Prevention and Treatment, Collaborative Innovation Center for Cancer Personalized Medicine, Nanjing Medical University, School of Public Health, Nanjing Medical University, Nanjing, China; ^6^ Department of Genetic Toxicology, The Key Laboratory of Modern Toxicology of Ministry of Education, Center for Global Health, School of Public Health, Nanjing Medical University, Nanjing, China; ^7^ The Affiliated Suzhou Hospital of Nanjing Medical University, Suzhou Municipal Hospital, Gusu School, Nanjing Medical University, Suzhou, China; ^8^ Department of Epidemiology, Center for Global Health, School of Public Health, Nanjing Medical University, Nanjing, China; ^9^ Duke Cancer Institute, Duke University Medical Center, Durham, NC, United States; ^10^ Department of Population Health Sciences, Duke University School of Medicine, Durham, NC, United States; ^11^ Yiwu Research Institute of Fudan University, Yiwu, Zhejiang, China; ^12^ Cancer Institute, Fudan University Shanghai Cancer Center, Shanghai Medical College, Shanghai, China

**Keywords:** gastrointestinal cancer, rare variants, cross-cancer susceptibility, ASSET analysis, pathway analysis

## Abstract

**Background:**

Cancers arising within the gastrointestinal tract are complex disorders involving genetic events that cause the conversion of normal tissue to premalignant lesions and malignancy. Shared genetic features are reported in epithelial-based gastrointestinal cancers which indicate common susceptibility among this group of malignancies. In addition, the contribution of rare variants may constitute parts of genetic susceptibility.

**Methods:**

A cross-cancer analysis of 38,171 shared rare genetic variants from genome-wide association assays was conducted, which included data from 3,194 cases and 1,455 controls across three cancer sites (esophageal, gastric and colorectal). The SNP-level association was performed by multivariate logistic regression analyses for single cancer, followed by association analysis for SubSETs (ASSET) to adjust the bias of overlapping controls. Gene-level analyses were conducted by SKAT-O, with multiple comparison adjustments by false discovery rate (FDR). Based on the significant genes indicated by SKATO analysis, pathways analysis was conducted using Gene Ontology (GO), the Kyoto Encyclopedia of Genes and Genomes (KEGG) and Reactome databases.

**Results:**

Meta-analysis in three gastrointestinal (GI) cancers identified 13 novel susceptibility loci that reached genome-wide significance (*P*_ASSET_< 5×10^-8^). SKAT-O analysis revealed *EXOC6, LRP5L* and *MIR1263/LINC01324* to be significant genes shared by GI cancers (*P*_adj_<0.05, *P*_FDR_<0.05). Furthermore, GO pathway analysis identified significant enrichment of synaptic transmission and neuron development pathways shared by all three cancer types.

**Conclusion:**

Rare variants and the corresponding genes potentially contribute to shared susceptibility in different GI cancer types. The discovery of these novel variants and genes offers new insights for the carcinogenic mechanisms and missing heritability of GI cancers.

## 1 Introduction

Gastrointestinal (GI) cancers represent the most commonly diagnosed cancer types globally, a group of cancers exhibiting the highest incidence burden (1). According to GLOBOCAN 2020, GI cancers account for approximately 18.8% of cancer incidents and 22.6% of cancer deaths worldwide, and contributing to major public health burdens (2). The incidence of GI cancers also varies by country and region. China has far more new cases of GI cancers than the rest of the world, accounting for 29.7% and 32.0% of all cancer cases and deaths in the region in 2020 (2, 3). The particular high incidents were seen in esophageal and gastric cancer; both accounted for about half of the new cases in the world in 2020 (esophageal cancer: 53.7%, gastric cancer: 44.0%) (2). Although the incidence of colorectal cancer in China is moderate comparing to high-incidence country, it is still higher than global average along with a rising trend over the past few years (4). It is established that genetic and lifestyle factors play vital roles in the risk of cancer. Family studies and population-based studies also revealed genetic components contributing to cancer susceptibility, possibly by modifying risk factors induced by environmental carcinogens or altering the functions of critical regulatory pathways (5).

It is estimated that approximately 5% of GI cancers incidents are attributed to inherited genetic mutations with familial aggregation characteristics (6), and an additional 20-25% are estimated to have hereditary components which yet to be established. Most of these cancers develop from sporadic events, with diverse arrays of genetic variations contributing to cancer susceptibility. Tremendous efforts have been done in the search for missing heritability; in particular genome-wide association studies (GWAS) and next-generation sequencing (NGS) technology have been performed to identify the effects of common and rare variants across various cancer types (6). GWASs have been instrumental in deciphering the effects of common variants (Minor Allele Frequency (MAF) > 1%) on the carcinogenesis of single cancer (7–9). However, the confirmed candidate causal variants so far only explain for a small proportion of the heritability. The effects of rare variants (MAF<1%) have been relatively less investigated. Based on the established heritability for GI cancers, more evidence suggests that rare variants exhibiting large effect sizes still remain to be discovered. These variants displaying high penetrance may have more profound clinical manifestations (10). The aggregation of rare variants in predisposition genes can lead to changes in gene expression or function (11), and altered functions in biological pathways may be pivotal in the carcinogenetic process of GI cancers.

Discerning the molecular mechanisms of sporadic GI cancers is complex. In addition to the effort in single cancer research, shared genetic risk factors have been reported in various cancer groups, and target genes with potential cross-cancer roles have been identified at specific genetic loci. For instance, shared mechanisms of carcinogenesis were reported in hormone-sensitive and obesity-related cancers (12, 13). Common variants in epigenetic and vitamin D metabolism genes were reported for the susceptibility of a subset of GI cancer (14, 15). A pan-cancer study suggested common genes were shared between 12 cancer types, including gastric cancer (16). The 9p21 region have been associated with multiple tumors including esophageal squamous cell carcinoma (ESCC) and endometrial cancer (17, 18). However, the effects of shared rare variants have been scarcely investigated in the literature. Based on this background, we conducted a cross-cancer study of rare variants by performing meta-analysis on the genotyping data from genome-wide association arrays on esophageal, gastric and colorectal cancers, and assess the aggregate effects of significant variants on the gene level. We hypothesize that these rare genetic variants may facilitate identification, at genome-wide significance, of risk loci shared among GI cancer, leading to the identification of novel susceptibility genes and pathways contributing to carcinogenesis, which may in turn explain part of the missing heritability.

## 2 Materials and methods

### 2.1 Study population

**Figure 1:**
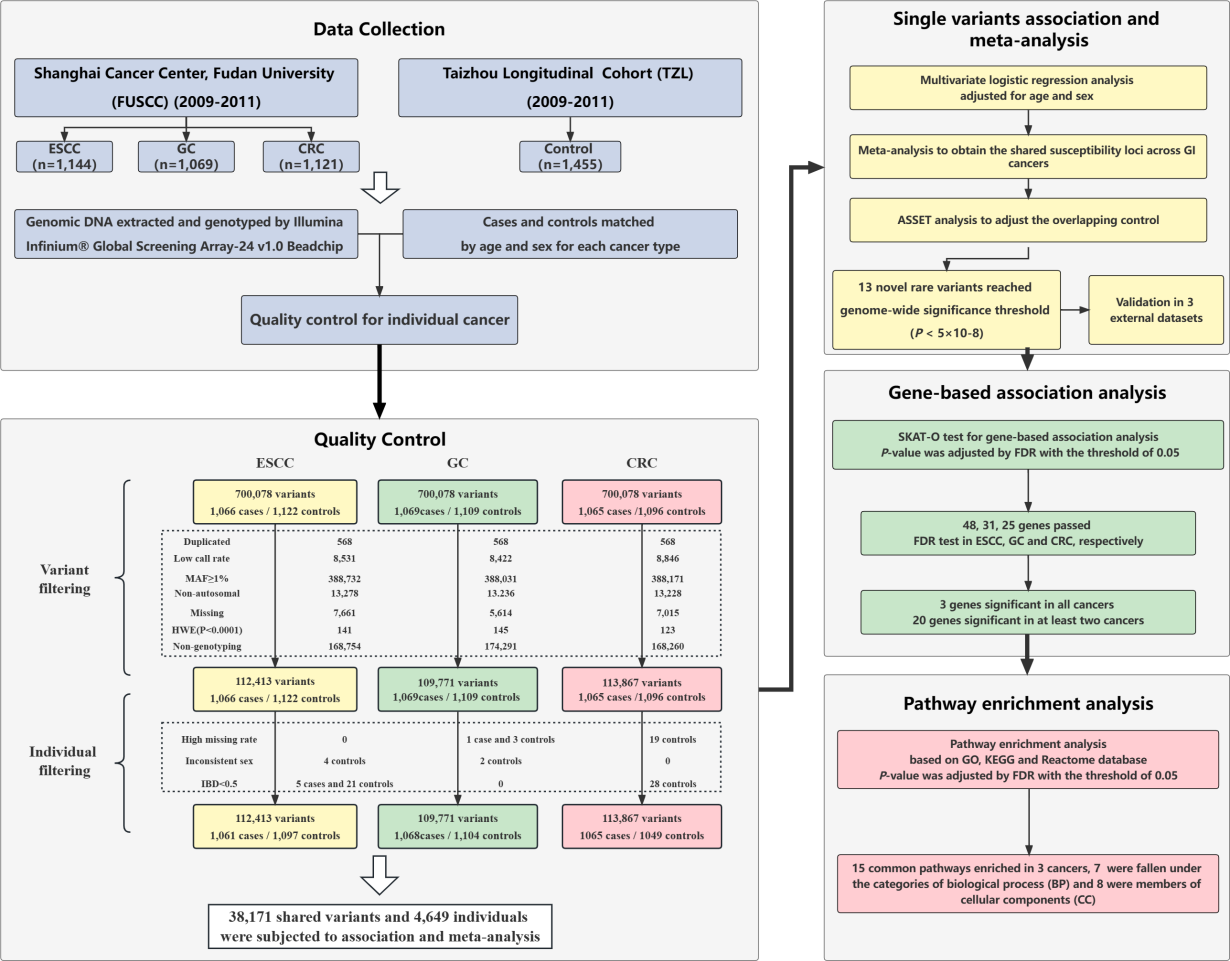
Schematic overview of cross-cancer analysis and quality control process.

The ESCC, GC, and CRC patients from Fudan University Shanghai Cancer Center (FUSCC) were derived from published studies (19), which were newly diagnosed and histopathologically confirmed between 2009 and 2011. The control population was enrolled at the same time from a cancer-free healthy Han individuals (n=1455) in Taizhou Longitudinal cohort (TZL) study from Eastern China (20). The inclusion criteria and exclusion criteria of patients and controls were described in detail previously (21–23), and patients of each cancer type were matched with the cancer-free controls by sex and age ( ± 5 years). After matching, 1,066 cases and 1,122 controls, 1,069 cases and 1,109 controls, and 1,065 cases and 1,096 controls were included in the ESCC, GC and CRC datasets, respectively (**Figure 1**). Blood samples from ESCC, GC, CRC patients and cancer-free controls were provided by the tissue banks of FUSCC and TZL studies, respectively. Each participant donated approximately 10 mL of peripheral blood, from which genomic DNA was extracted. For validation, three independent datasets were recruited, which included Beijing ESCC GWAS dataset (cases/controls:2,031/2,044), Chinese GC meta-GWAS (cases/controls:10,254/10,914), Nanjing CRC GWAS (cases/controls: 1,316/2,207). The details of the external validation population have been described in previous studies (8, 24, 25). All participants provided written informed consent for scientific research use of their biological samples and demographic information, including age, sex and smoking. The study protocol was approved by the Institutional Ethics Review Board of FUSCC.

### 2.2 Genome-wide association scanning and quality control

Genomic DNA was extracted from the blood samples of patients and controls, and genome-wide genotyping was performed by the Infinium^®^ Global Screening Array-24 v1.0 Beadchip (Illumina, US). Quality control (QC) of the raw genotyping results was conducted in the case-control dataset of each cancer type. The exclusion criteria of SNPs were set as follows: 1) Duplicated variants; 2) Missing rate over 5% between cases and controls; 3) MAF ≥ 1%; 4) SNPs not mapped to autosomal chromosomes; 5) SNPs with genotyping frequency = 0; 6) Deviation from Hardy-Weinberg Equilibrium (HWE) test (*P*< 1x10^-4^). The exclusion criteria of ineligible individuals are described as follows: 1) Inconsistent sex between determined genotypes and clinically derived information; 2) First-degree relatives (IBD< 0.5); 3) Missing genotype type > 5%. After QC, 2,158 individuals (1,061 cases/1,097 controls) and 112,413 variants in ESCC dataset, 2,172 individuals (1,068 cases/1,104 controls) and 109,771 variants in GC dataset, 2,114 individuals (1,065 cases/1,049 controls) and 113,867 variants in CRC dataset were retained and subjected to further analysis. The details of the QC process are illustrated in the study flow chart (**Figure 1**).

### 2.3 Variant classification and annotation

After QC, 38,171 genetic variants (MAF< 0.01) were found to be shared among the 3 cancer types. ANNOVAR software was used to annotate these rare variants by their location and characteristics (26). Overall, 35,923 of 38,171 shared variants in three cancer types were successfully annotated and classified by ANNOVAR. We divided the variants into nine categories: missense mutation, nonsense/nonstop mutation, intergenic region mutation, intronic mutation, ribonucleic acid (RNA) mutation, 3’ untranslated region (3’UTR) mutation, 5’ untranslated region (5’UTR) mutation, splice site mutation, in-frame/frame-shift insertion/deletion mutation. All variants were genotyped by GSA BeadChip array, which is comprised of SNPs or indels. The results of rare variant classification determined by ANNOVAR are presented in **Supplementary Figure 1**.

### 2.4 Single variant association analysis

First, logistic regression models were applied in 3 cancer datasets separately to assess the association between single variant and the risk of ESCC, GC and CRC. Odds ratio (OR) with 95% confidence interval (CI) and *P*-values were calculated by additive model adjusted for sex and age. Then meta-analysis of the individual cancer results was conducted to determine the pooled association across these three cancer types, with the significance criteria of genome-wide threshold (*P*<5×10^-8^), consistency in risk alleles and odds ratio. The fixed effect model was applied if SNPs passed Cochran’s Q test (Q > 0.1), otherwise random effect models were considered. The association results of individual cancer and meta-analysis were presented by Manhattan plot. Genomic inflation statistics (λ) were calculated and visualized by quantile-quantile (Q-Q) plot to evaluate population stratification in each dataset. To account for the correlation between datasets due to overlapping controls, we applied Association analysis for SubSETs test (ASSET), which is a subset-based approach to determine the best subset of subjects with maximized test statistics for meta-analysis (27). In our study, all significant SNPs passed Cochran’s Q test and showed consistent direction of association in each cancer; therefore, one-sided ASSET analysis was conducted with a fixed-effect model (28). In order to identify the effects of gender, age, and smoking on the results of the single variant, we further stratified the population by men, women, age ≤60, age > 60, never smoking and ever smoking, and performed logistic regression analysis in each subgroup. The significant variants identified by meta-analysis were further validated in external datasets from previous reported GWAS.

### 2.5 Gene-based association analysis

Due to the fact that the MAF of rare variants in the population is very low, affected individuals may have different mutation sites or frequencies in a particular genetic region of interest. To account for the low efficiency that traditional single variants association analysis may incur, rare variants can be collapsed or compressed into a set and then examined for inter-set frequency differences between case and control groups (29). A few statistical tests have been developed, including the burden test and Sequence Kernel Association Test (SKAT) (30). The method selection depends on the number of rare mutations with the same direction of action. However, since the genetic model in complex diseases is unknown in practice, it is challenging to select the optimal application method. The optimal sequence kernel association test (SKAT-O) overcomes this problem by including the correlation matrix of the relationship structure of rare variants in the SKAT test (31), with the formula listed as follows:


$${\text{Q}}_{\text{ρ}}=\text{ρ}{\text{Q}}_{\text{b}}+(1-\text{ρ}){\text{Q}}_{\text{s}},0\leq \text{ρ}\leq 1$$

Q_b_ and Q_s_ are the score statistics of the burden test and SKAT test under the null model. The correlation matrix contains a parameter ρ, and mimics the burden test (when ρ= 1) or general SKAT (when ρ= 0). Different genetic structures of different traits correspond to different optimal ρ values. In practical application, ρ is obtained through the test procedure, and the weighted average value of SKAT and burden test statistics is calculated. In this study, the SKAT-O test was applied for gene-based association analysis by using the R package ‘SKAT’ (V2.0.1). For the loci shared by three cancer types, two or more loci located in the same genetic region were combined into a SET (genes) (32). Additionally, the obtained SKAT-O *P*-value was adjusted by FDR to control the type I error. Genes with FDR-adjusted *P*-values (*P_adj_*) of less than 0.05 were considered significant.

### 2.6 Pathway enrichment analysis

In order to determine the underlying biological pathways affected by the significant genes identified by SKAT-O analysis, enrichment analysis was performed by Gene Ontology (GO), the Kyoto Encyclopedia of Genes and Genomes (KEGG) and Reactome databases by R package ‘clusterProfiler’ (V4.4.4). For intergenic variants, both annotated genes were included in pathway analysis with subsequent FDR correction. The total gene lists contained 961, 959 and 1,011 genes in ESCC, GC and CRC dataset, respectively. Due to the presence of overlapping genes in common pathways, *P*-values were adjusted by FDR to control for a low proportion of false positives with the threshold of 0.05. In addition, we calculated Fold Enrichment (FE) by dividing Gene Ratio by Background Ratio to normalize the size of gene set. The shared pathways were identified after pooling the pathway enrichment results of these three GI cancers. Within the shared pathways, we discarded the ‘integral component of postsynaptic density membrane’ and ‘intrinsic component of postsynaptic density membrane’ because they represent protein topology, not a cellular component, and were already obsoleted by the newest vision of the database. For pathways which are subsets of larger pathways, only the larger pathways were retained in our results, the subset pathways were discussed when we explored potential mechanisms of association between shared pathways and GI cancer risk.

### 2.7 Statistical analysis

The comparison of demographic characteristics between the case and control groups was performed by Chi-square test or two independent sample Wilcoxon rank sum test. Variant filtering and quality control were conducted by PLINK (V1.9) and R software (V4.2.0). For single variant association analysis, multivariate logistic analyses were performed by PLINK 1.9 adjusted for sex and age, followed by one-sided ASSET using R package ‘ASSET’ (V2.14.0). Meta-analysis was performed using fixed-effect model followed by Cochran’s Q test. Genome-wide statistical significance was set at *P*-value of 5x10^-8^. Manhattan plots and Q-Q plots of each dataset were carried out by R package ‘qqman’ (V0.1.8). Functional annotation of variants was carried out by ANNOVAR based on human genome hg19 coordinates.

## 3 Results

### 3.1 Population characteristics of study subjects

After quality control of the recruited individuals and genetic data, three case-control datasets of esophageal squamous cell carcinoma (ESCC), gastric cancer (GC) and colorectal cancer (CRC) were included in the study (**Figure 1**). **Table 1** summarizes the general demographic characteristics of the study samples included in the three cancer types. The age and sex distribution of cases and controls in the three cancer types were comparable (*P* > 0.05). In the ESCC and GC datasets, over half of the subjects were under 60 years of age, whereas over 50% of the subjects in CRC dataset were over 60 years old. All three datasets showed a higher percentage of male. The distribution of smoking status was significantly different between cases and controls in all three datasets (*P<* 0.001). More cases in the ESCC dataset were current smokers (57.7%) compared to the control group (36.6%). The majority of the GC and CRC samples were non-smokers, and 38.5% of the patients in the GC’s case group were either smokers or ever-smokers compared to 36.6% in the control group. In the CRC dataset, only 16.2% of the patients were smokers, whereas 32.3% of control samples have smoking history.

**Table 1:** Characteristics of the study population.

Variables		ESCC			GC			CRC		
		Cases	Controls	*P^a^*	Cases	Controls	*P^a^*	Cases	Controls	*P^a^*
No. of Patients		1061	1097		1068	1104		1065	1049	
Age	≤60	533(50.2%)	495(45.1%)	0.09	573(53.7%)	580(52.5%)	0.46	510(47.9%)	495(47.2%)	0.55
	>60	528(49.8%)	602(54.9%)		495(46.3%)	524(47.5%)		555(52.1%)	554(52.8%)	
Sex	Male	868(81.8%)	864(78.8%)	0.08	758(71.0%)	778(70.5%)	0.8	670(62.9%)	671(64.0%)	0.39
	Female	193(18.2%)	233(21.2%)		310(29.0%)	326(29.5%)		395(37.1%)	378(36.0%)	
Smoking Status^b^	Non-smoker	358(33.7%)	587(53.5%)	<0.001	651(61.0%)	683(61.9%)	<0.001	892(83.8%)	613(58.4%)	<0.001
	Smoker	612(57.7%)	402(36.6%)		68(6.4%)	392(35.5%)		173(16.2%)	304(29.0%)	
	Ever-smoker	4(0.4%)	23(2.1%)		343(32.1%)	12(1.1%)		0(0.0%)	33(3.2%)	
	Missing	87(8.2%)	85(7.8%)		6(0.6%)	17(1.5%)		0(0.0%)	99(9.4%)	

Two independent sample Wilcoxon rank sum test was used for age variables in ESCC, GC and CRC datasets. ^a^ χ2 test was used for gender and smoking status variables. ^b^ Non-smoker: never smoke; Smoker: Smoking, and still smoking; Ever-smoker: used to smoke, but quitted.

ESCC, Esophageal Squamous Cell Carcinoma; GC, Gastric Cancer; CRC, Colorectal Cancer.

### 3.2 Meta-analysis of single variant across GI cancers

**Figure 2:**
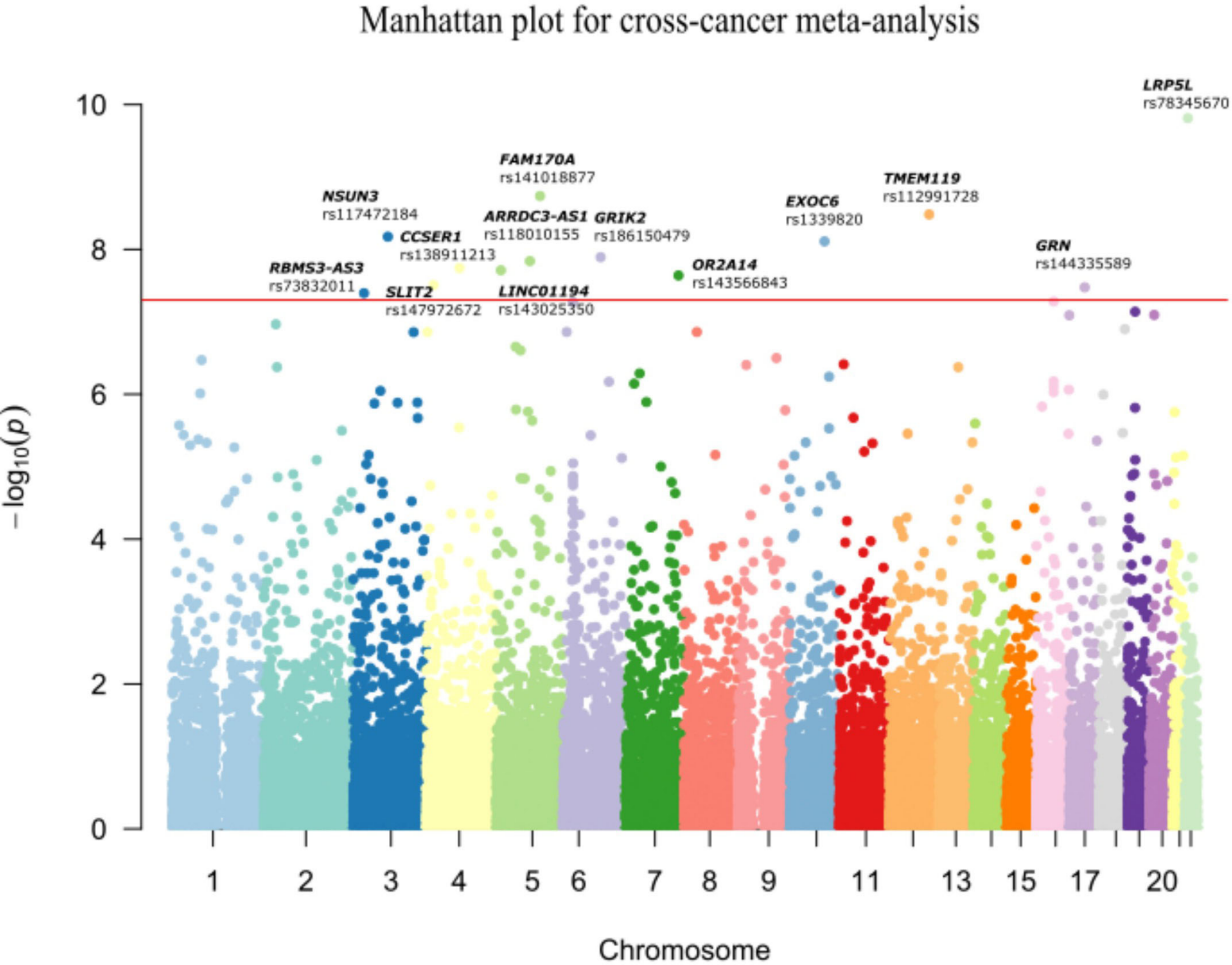
Manhattan plot for cross-cancer meta-analysis. The meta-analysis revealed 13 shared variants passed the genome-wide threshold of 5×10^-8^in ESCC, GC and CRC. The x-axis denotes the chromosome location of the variants; the y-axis represents the -log_10_(*P*_meta_) value of each variant. The rsID of the significant SNPs and the corresponding genes are annotated.

Multivariate logistic regression analysis in ESCC, GC and CRC datasets was performed separately with adjustment for sex and age. A total of 29, 28 and 10 variants were significantly associated with ESCC, GC and CRC risk, respectively (*P<* 5x10^-4^) (**Supplementary Figure 2**). The Q-Q plots of each cancer type showed modest deviations of observed *P*-value from expected *P*-value (**Supplementary Figure 3**). (λ_ESCC_=1.029, λ_GC_=1.068 and λ_CRC_=1.069). In order to assess the shared susceptibility loci across GI cancers, we conducted a meta-analysis to combine the association results of three cancers. Based on one-sided Association analysis based on SubSET (ASSET) analysis (**Table 2**; **Figure 2**; **Supplementary Table 1**), 13 novel rare variants were found to reach the genome-wide significance threshold (*P*_ASSET_< 5×10^-8^), with no pleiotropy or high homogeneity across three cancer types (same direction of effect and Q > 0.1). Of the 13 variants, 8 were likely to be risk alleles (OR >1), while 5 may confer protective effects (OR<1). The most significant variant was *LRP5L*/rs78345670 (A→G) (19), which was also the top hit variant in GC (OR (95% CI) = 11.13 (3.32-37.32), *P* = 9.50×10^-5^) and CRC dataset [OR (95% CI) = 6.73 (2.62-17.30), *P* = 7.67×10^-5^]. Exonic variant *TMEM119*/rs112991728 (G→A) [OR (95% CI) = 0.09 (0.04-0.19), *P_ASSET_* = 3.29×10^-9^) was identified as the most significant protective variant. In the ESCC dataset, intergenic variant rs117472184 in *NSUN3* (G→A) showed the most significant association with ESCC risk [OR (95% CI) = 0.09 (0.03-0.28), *P* = 5.00×10^-5^].

**Table 2:** Meta-analysis and one-sided ASSET analysis of ESCC, GC and CRC datasets.

Chr.	rsID	Nearest Gene	Location	Allele^a^	MAF^b^	MAF^c^	ESCC		GC		CRC		Meta-analysis		One-sided ASSET Analysis	
							OR(95% CI)^d^	*P^d^*	OR(95% CI)^d^	*P^d^*	OR(95% CI)^d^	*P^d^*	OR (95%CI)	*P*_Meta_	OR (95%CI)	*P*_ASSET_
22	rs78345670	*LRP5L*	intronic	G	0.014	0.002	10.46 (3.18-34.46)	1.14E-04	11.13 (3.32-37.32)	9.50E-05	6.73 (2.62-17.30)	7.67E-05	8.73 (4.64-16.42)	1.75E-11	8.63 (4.46-16.71)	1.54E-10
5	rs141018877	*FAM170A*	intergenic	G	0.015	0.004	8.38 (2.95-23.79)	6.53E-05	3.42 (1.59-7.37)	1.68E-03	3.35 (1.64-6.82)	9.03E-04	4.05 (2.54-6.47)	4.26E-09	4.45 (2.74-7.24)	1.83E-09
12	rs112991728	*TMEM119*	exonic	A	0.001	0.012	0.10 (0.03-0.32)	1.15E-04	0.10 (0.03-0.32)	1.66E-04	0.10 (0.03-0.34)	1.99E-04	0.10 (0.05-0.20)	5.83E-11	0.09 (0.04-0.19)	3.29E-09
3	rs117472184	*NSUN3*	intergenic	A	0.002	0.015	0.09 (0.03-0.28)	5.00E-05	0.07 (0.02-0.31)	3.84E-04	0.25 (0.10-0.61)	2.22E-03	0.14 (0.08-0.27)	2.64E-09	0.15 (0.08-0.28)	6.67E-09
10	rs1339820	*EXOC6*	3’-UTR	A	0.012	0.002	5.74 (1.97-16.71)	1.36E-03	15.72 (3.71-66.67)	1.86E-04	5.84 (2.01-16.97)	1.18E-03	7.17 (3.67-14.01)	7.89E-09	7.65 (3.84-15.27)	7.75E-09
6	rs186150479	*GRIK2*	intergenic	A	0.002	0.014	0.13 (0.05-0.36)	9.52E-05	0.14 (0.04-0.48)	1.70E-03	0.11 (0.03-0.36)	2.81E-04	0.13 (0.07-0.24)	6.38E-10	0.12 (0.06-0.25)	1.28E-08
5	rs118010155	*ARRDC3-AS1*	intergenic	G	0.013	0.003	6.83 (2.65-17.61)	7.00E-05	6.57 (2.24-19.26)	6.05E-04	3.53 (1.60-7.78)	1.78E-03	5.04 (2.97-8.54)	2.05E-09	5.02 (2.87-8.76)	1.45E-08
4	rs138911213	*CCSER1*	intronic	A	0.014	0.004	5.83 (2.24-15.15)	3.02E-04	4.33 (1.96-9.57)	3.00E-04	3.31 (1.57-7.01)	1.75E-03	4.18 (2.61-6.72)	3.15E-09	4.20 (2.55-6.93)	1.81E-08
5	rs143025350	*LINC01194*	intergenic	A	0.001	0.014	0.03 (0.00-0.25)	8.53E-04	0.11 (0.04-0.37)	2.87E-04	0.19 (0.07-0.48)	4.73E-04	0.13 (0.06-0.26)	5.09E-09	0.13 (0.06-0.27)	1.94E-08
7	rs143566843	*OR2A14*	intergenic	A	0.002	0.014	0.10 (0.03-0.33)	1.57E-04	0.19 (0.08-0.48)	4.23E-04	0.27 (0.12-0.61)	1.88E-03	0.19 (0.11-0.34)	3.93E-09	0.17 (0.09-0.32)	2.29E-08
4	rs147972672	*SLIT2*	intronic	C	0.013	0.003	4.73 (1.94-11.52)	6.15E-04	4.81 (2.07-11.15)	2.55E-04	3.51 (1.51-8.18)	3.65E-03	4.29 (2.62-7.05)	8.25E-09	4.43 (2.61-7.49)	3.10E-08
17	rs144335589	*GRN*	intergenic	A	0.013	0.003	5.25 (2.00-13.76)	7.57E-04	5.09 (2.08-12.47)	3.69E-04	3.82 (1.66-8.83)	1.69E-03	4.60 (2.75-7.72)	6.83E-09	4.69 (2.71-8.12)	3.34E-08
3	rs73832011	*RBMS3-AS3*	ncRNA_intronic	A	0.012	0.004	4.60 (1.74-12.21)	2.16E-03	4.10 (1.74-9.66)	1.25E-03	3.88 (1.77-8.47)	6.87E-04	4.13 (2.51-6.79)	2.23E-08	4.48 (2.62-7.64)	4.02E-08

The table presents the meta-analysis and ASSET analysis results of ESCC, GC and CRC dataset for newly identified loci reaching genome-wide significance (P<5x10-8). P-values of meta-analysis were derived from fixed-effects models. One-sided ASSET analysis was used to correct the false positive rate caused by overlapping controls in three datasets. ^a^ Effect Allele; ^b^ MAF of cases in all datasets; ^c^ MAF of controls in all datasets; ^d^ Multivariate logistic regression in additive models adjusted for sex and age.

Chr, chromosome; MAF, minor allele frequency; OR, odds ratio; ASSET, Association analysis based on SubSETs.

The significant loci in the meta-analysis were further validated in three external datasets (**Supplementary Table 5**). Three variants were successfully replicated in the ESCC dataset, and 2 variants were found significantly associated with GC and CRC risks, respectively (*P*<0.05). However, no common variant passed the significant threshold (set as 0.05) in all three validation datasets. Two variants were replicated in two validation populations. *EXOC6*/rs1339820 – a significant SNP in GC validation [OR (95% CI) = 1.28 (1.03-1.60), *P* = 0.03], was also significantly associated with CRC in validation [OR (95% CI) = 0.44 (0.24-0.81), *P* = 0.01]. *ABCC11*/rs75797074 A allele showed protective effect with GI cancer risk in ESCC and CRC validation datasets while the results of ASSET meta-analysis indicated that it was a risk allele for GI cancer initiation [OR (95% CI) = 4.51 (2.62-7.77), *P_ASSET_* = 5.23×10^-8^].

### 3.3 Stratified analysis of single variant

The results of stratified analysis showed consistent effects in most subgroups in each cancer type, although the *P*-values of some variants were not significant due to insufficient number of samples (**Supplementary Tables 2–4**). After stratifying the population by gender, age and smoking status, the effects of some GI cancer-associated variants seem to differ across different cancers. Age seems to have a specific effect on the ESCC and GC for two variants. For holders of risk allele *LRP5L*/rs78345670-G, people under age of 60 may have a higher risk for ESCC [OR (95% CI) =13.50 (1.77-103.10), *P*=0.012], whereas older people (>60 years old) may be more susceptible to GC [OR (95% CI) =18.54 (2.46-139.90), *P*=0.005]. The opposite effect of age is seen in *EXOC6*/rs1339820-A holders and risk of ESCC and GC. For holders of the *SLIT2*/rs147972672-C allele, females have a 2.5-fold increased risk of ESCC compared to males, whereas males are at higher risk for GC. For bearers of *RBMS3-AS3*/rs73832011-A, the risk for all three GI cancers is only significant in non-smokers. The modifying effect of demographic or lifestyle factors found above is based on the assumption of holding a single variant, for people holding more than one risk variants, this modifying effect may change.

### 3.4 Gene-based rare variant analysis

Based on the principle of the Optimal Sequence Kernel Association Test (SKAT-O) analysis, 38,171 shared cross-cancer loci were merged into SETs (genes), whereas two or more loci located in the same gene region were merged into a set, and 6,741 sets spanning 28,152 loci were constructed. After FDR correction, 48, 31 and 25 genes were found to be significant in ESCC, GC and CRC, respectively, of which 20 were significant in at least two cancer types (**Table 3**). Among which three genes, *EXOC6* (*P_adj|ESCC_* =7.30×10^-3^, *P_adj|GC_* =6.00×10^-4^, *P_adj|CRC_* =2.6×10^-2^), *LRP5L* (*P_adj|ESCC_* =2.70×10^-3^, *P_adj|GC_* =5.70×10^-3^, *P_adj|CRC_* =1.65×10^-2^), *MIR1263/LINC01324*(*P_adj|ESCC_* =3.22×10^-2^, *P_adj|GC_* =4.36×10^-2^, *P_adj|CRC_* =3.26×10^-2^) were statistically significant in all three cancer types. In addition, two included SNPs *EXOC6/*rs1339820 and *LRP5L*/rs78345670 were also significant in the single variant meta-analysis. *ABCC11* showed significant in GC and CRC dataset (*P_adj|GC_* =4.77×10^-2^, *P_adj|CRC_* =2.46×10^-2^) while the variant rs75797074 located in *ABCC11* nearly passed ASSET analysis with the *P*-value of 5.23×10^-8^ (**Supplementary Table 1**). Detailed information of the SNPs included in these 20 genes is summarized in the **Supplementary Table 6**.

**Table 3:** Significant genes associated with GI cancers by SKAT-O analysis.

Gene region	Chr. position^a^	cMAF	No. SNPs^b^	ESCC		GC		CRC		Significance in cancer^c^
				*P-*value	FDR	*P-*value	FDR	*P-*value	FDR	
*LRP5L*	22q11.23	0.005	2	3.65E-06	2.70E-03	3.36E-06	5.70E-03	8.10E-06	1.65E-02	1
*EXOC6*	10q23.33	0.005	3	1.76E-05	7.30E-03	1.01E-07	6.00E-04	2.19E-05	2.46E-02	1
*MIR1263/LINC01324*	3q26.1	0.028	5	2.10E-04	3.22E-02	9.13E-05	4.36E-02	5.32E-05	3.26E-02	1
*EPDR1/STARD3NL*	7p14.1	0.005	2	1.41E-04	2.57E-02	6.91E-06	7.80E-03	1.70E-02	3.34E-01	2
*LINC01288/UNC5D*	8p12	0.023	4	1.86E-04	3.05E-02	1.83E-04	4.77E-02	8.96E-04	9.74E-02	2
*LINC01683/LINC02573*	21q21.1	0.041	6	1.72E-05	7.30E-03	1.15E-04	4.36E-02	1.54E-03	1.24E-01	2
*LINC02156/RPAP3*	12q13.11	0.007	4	2.49E-05	7.30E-03	2.13E-04	4.78E-02	6.49E-03	2.40E-01	2
*LRP3*	19q13.11	0.016	2	3.68E-07	8.00E-04	2.10E-04	4.78E-02	1.87E-01	6.84E-01	2
*MIR548XHG/LINC01683*	21q21.1	0.044	13	5.10E-07	8.00E-04	1.90E-04	4.77E-02	1.05E-03	1.09E-01	2
*NAALADL2*	3q26.31	0.053	13	7.48E-07	8.00E-04	1.75E-07	6.00E-04	7.54E-04	9.08E-02	2
*NETO1*	18;18 E4	0.010	2	4.56E-05	1.14E-02	1.52E-04	4.74E-02	2.48E-03	1.59E-01	2
*NSUN3/MIR6730*	3q11.2	0.039	8	8.76E-07	8.00E-04	1.16E-04	4.36E-02	1.26E-02	3.04E-01	2
*SEPTIN9*	17q25.3	0.023	4	1.01E-05	5.70E-03	6.98E-05	3.92E-02	1.65E-01	6.67E-01	2
*TASP1*	20p12.1	0.026	6	8.24E-07	8.00E-04	8.78E-06	8.50E-03	4.68E-04	7.69E-02	2
*TPSD1/UBE2I*	16p13.3	0.013	2	4.56E-06	3.00E-03	2.07E-04	4.78E-02	2.49E-01	7.42E-01	2
*ZNF737*	19p12	0.003	2	8.14E-05	1.58E-02	6.86E-05	3.92E-02	2.03E-03	1.46E-01	2
*ABCC11*	16q12.1	0.023	8	6.61E-04	6.55E-02	1.91E-04	4.77E-02	3.29E-05	2.46E-02	3
*MYCBP2*	13q22.3	0.013	3	1.99E-02	3.48E-01	2.23E-04	4.84E-02	1.50E-04	4.74E-02	3
*FAM170A/PRR16*	5q23.1	0.038	12	4.88E-06	3.00E-03	1.38E-03	1.24E-01	2.83E-05	2.46E-02	4
*SLC22A2*	6q25.3	0.012	2	1.50E-05	7.20E-03	5.66E-04	7.57E-02	2.42E-05	2.46E-02	4

a Chromosome Build GRCh37;

b No. SNPs =Total number of SNPs binned in the gene;

c 1= significant genes in three cancers; 2= significant genes in ESCC and GC; 3= significant genes in GC and CRC; 4= significant genes in ESCC and CRC.

cMAF, cumulative minor allele frequency.

### 3.5 Pathway enrichment analysis

**Figure 3:**
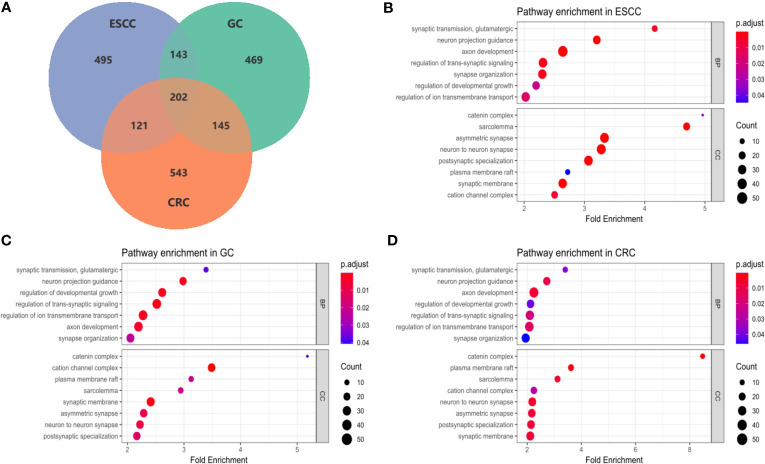
Enrichment analysis of common GI pathways in ESCC, GC and CRC. **(A)** The Venn diagram showed the numbers of potential susceptibility genes included in pathway enrichment analysis in ESCC, GC and CRC datasets. Overall, 202 genes were common in 3 cancer type, additionally, 143 genes were common in ESCC and GC, 121 genes were common in ESCC and CRC, and 145 genes were common in GC and CRC; **(B–D)** Bubble plots of pathway enrichment in ESCC, GC and CRC. Among 15 common pathways, 7 pathways were under the categories of biological process (BP), and 8 pathways belonged to cellular components (CC).

Significant genes in each cancer type identified by SKAT-O were subjected to pathway enrichment analysis based on GO, KEGG and Reactome databases. FDR test was conducted in pathway analysis rather than SKAT-O to avoid over-adjustment. As a result, GO enrichment analysis identified 180, 168, 115 pathways in ESCC, GC and CRC datasets, in which 24 were common across cancers (**Supplementary Tables 7–10**). In contrast, no significant pathways passed FDR tests in the KEGG and Reactome databases. After filtrating 7 subset pathways and 2 obsoleted pathways, we found 15 pathways which were shared in 3 cancers (**Figure 3**), 7 pathways were fallen under the categories of biological process (BP) and 8 pathways belonged to cellular components (CC). The majority of the pathways represent neurological functions and structures, indicating the potential association between neurogenesis and cancer risks. Among the BP pathways, ‘glutamatergic synaptic transmission’ showed the largest fold enrichment (FE) in all three cancers, while ‘axonogenesis’ had the most significant adjusted *P*-value and most enriched genes in ESCC and CRC datasets. According to the GO database, ‘axonogenesis’ is a subset of the pathway ‘axon development’, in that case, we included the larger pathways in the bubble diagram. Within the CC category, ‘catenin complex’ presented the largest FE in all three cancers (FE_ERCC_ = 4.96, FE_GC_ = 5.18, FE_CRC_ = 8.48), which was also the most significant pathway in CRC dataset.

## 4 Discussion

Most of the GI cancers share similar multistep carcinogenic processes, therefore exploring shared genetic susceptibility factors can broaden our understanding of the underlying mechanisms in GI cancers. Previous genome-wide association studies focusing on common variants (MAF > 0.01) revealed a handful of loci which associated with individual types of cancer, leaving a considerable proportion of genetic susceptibility remain to be discovered. We hypothesized that parts of the susceptibility may be explained by rare variants with larger effect sizes, and the cross-cancer design may increase the statistical power to detect shared genetic features. This analytical strategy was implemented through meta-analysis along with ASSET method of single variants and supplemented with combined gene-based approach. The biological significance of the identified genes was further explored by pathway enrichment analysis. As a result, we identified 13 novel independent genetic variants associated with GI cancer susceptibility, 20 potential susceptibility genes shared by at least two GI cancers by SKAT-O analysis (3 genes shared by all three cancer types), and 15 pathways that were significantly enriched within three GI cancers.

In the single variant cross-cancer meta-analysis, the most significant SNP rs78345670 resides in the intronic region of *LRP5L*, which is also one of the most significant genes in SKAT-O analysis shared by all three cancers. Another variant rs1339820 located in the 3’-UTR region of *EXOC6* which was also a member of shared associated genes in GI cancers. This variant was also significant in GC and CRC validation datasets, although only the OR orientation in GC was consistent with the original meta-analysis. *ABCC11/*rs75797074, a significant SNP associated with risk of all three cancers (yet not passed the ASSET testing threshold), was successfully validated in the CRC and ESCC datasets (**Supplementary Table 5**). These three variants or SNPs in their corresponding LD blocks were not previously associated with GI cancer risk. *EXOC6* has been shown to be a predictor of breast cancer in previous study (33), but its role in GI cancers has not been reported. Taken together, *EXOC6*/rs1339820 and *ABCC11*/rs75797074 showed some cross-cancer susceptibility in CG and CRC, and *LRP5L* maybe a potential susceptibility gene in GI cancers.

Furthermore, gene-based association analysis revealed 20 genes that were shared by at least two GI cancers by SKAT-O analysis. Among them three genes were implicated in all three cancer types, two of which correspond to the significant SNPs in the single variant analysis. LDL Receptor Related Protein 5 Like (*LRP5L*) is a key gene involved in the regulation of Wnt signaling pathway, which plays an important role in tumor progression (34). Abnormal Wnt signaling pathway has been identified in various types of cancer, and it has been reported to be involved in maintaining abnormal proliferation of gastric cancer (35). It is also reported that *TMEM119* that encode membrane proteins may regulate Wnt signaling pathway according to previous studies (36, 37). However the potential role of these genes in GI cancers were not thoroughly studied. We have conducted intersection search on Pubmed for all significant genes found in our study, and found *LPR5L*, *TMEM119* and *SLIT2* have been reported in other non-GI cancer types. *TMEM119* was observed to be overexpressed in ovarian cancer (OV) tissues and associated with poor survival in OV patients. Overexpression of this gene also promoted proliferation, invasion, and migration in OV cells (38). Similarly in osteosarcoma, *TMEM119* was connected with tumor size, clinical stage and overall survival time, and associated with cell cycle, metastasis, apoptosis as well as TGF-β signaling in osteosarcoma cell lines (39). *SLIT2* was reported to regulate breast tumor growth and metastasis by blocking the expansion of tumor vasculature (40), and was also reported to be a predictive marker for thyroid cancer (41). Abnormal expression of *LPR5L* has been reported in breast cancer and pancreatic cancer (42, 43). Therefore, *LPR5L, TMEM119* and *SLIT2* may confer some universal onco-susceptibility, while other identified genes were mainly related to GI cancer susceptibilities based on our results.

Pathway enrichment analysis indicated major pathways related to synaptic transmission, axon development, catenin complex and regulation of ion transmembrane transport in the carcinogenesis of three GI cancers. Different groups of metabotropic glutamate receptors (mGluRs) modulate part of GI tract selectively (44), among which group III mGluRs was implicated in CRC risk and considered as a prognostic marker in CRC (45). Our findings may extend its involvement in the carcinogenic process of GI cancers other than CRC. *GRIK2* (glutamate ionotropic receptor kainate type subunit 2) – one of the susceptibility genes indicated by single variant analysis, acts as a member of glutamatergic synaptic transmission pathway which may contribute to GI cancers carcinogenesis by regulating glutamate receptor in synapse (46). Based on literature search, *LRRN4* and *NLGN1* in the nerve or glutamatergic synapses complex were found to promote CRC progression. The transmembrane protein Neuroligin 1 (*NLGN1*) is reported to interact at the synapse with the tumor suppressor Adenomatous Polyposis Coli (APC), which is intensively involved in the pathogenesis of CRC and is a key player in the WNT/β-catenin pathway (47). As for the potential correlation between synapse and carcinogenesis, several researchers found that tumor cells can form pseudo-tripartite synapses with neurons to increase tumor growth (48–50). Genetic alterations in non-neural/neural synapse systems were reported to contribute to the development of ESCC (51). The synaptic adhesion-like molecule (SALM) was found to be a potential prognostic biomarker in GC patients (52). Former study showed that the hypermethylation silencing of *GRIK2* results in decreased colony formation and invasion, in gastric cancer cells (46). Their research indicated that ionotropic glutamate receptors were related to the development and progression of some GI cancers. In the pathway analysis, a total of 29 and 27 genes identified by SKAT-O were enriched in glutamatergic synapse pathway in ESCC and CRC (**Supplementary Tables 7–9**). Taken together it is plausible that these genes may contribute to GI cancers carcinogenesis by regulating glutamate receptor in synapse. According to the results of our pathway enrichment analysis, *GRIK2* participate in the ‘cation channel complex’ and ‘regulation of trans-synaptic signaling’ pathway. Therefore, we speculate that glutamate receptors may contribute to GI cancer risks mediated by crosstalk of various pathways related to synaptic transmission, organization and trans-membrane transport. Taken together, our research may provide the evidence of the association between these signaling pathways and GI cancer risks.

Neuron density is generally regarded higher in tumor tissues than normal tissues (53), and neuron development is considered as a common characteristic in tumor microenvironment; hence cancer-specific neurogenesis may promote cancer growth (54). *SLIT2* might mediate the process of axon guidance by attracting or repelling developing axons and migrating neurons (55). In our research, ‘axon development’, and its subgroups ‘axonogenesis’ and ‘axon guidance’ were significantly enriched in all three GI cancers. Consistent with our findings, a miRNAs-based study suggested that axon guidance, targeted by dysregulation of miRNAs, may mediate the effects of oncogenes in gastric, colorectal and liver cancers (56). Moreover, the interaction between axon guidance and the corresponding receptors plays a vital role in the formation of malignancies by regulating vascularization, cell survival, apoptosis and cell migration (57). To this end, we provide further evidence for the potential relationship between neuron development and the carcinogenic progress of GI cancers.

There are several limitations to our study. First, due to the retrospective nature of our patient cohort, missing information may affect the accuracy of our multivariate logistic regression analysis, which only adjusted for age and sex. Important lifestyle factors such as drinking cannot be assessed. Second, the sample size of each cancer type was relatively small, with the overlapping control population. Although we tried to address this potential issue by using the ASSET test, this may still result in limited statistical power. However, the research interest of cross-cancer susceptibility has allowed us to focus only on the shared association through the meta-analysis. Last, our study population come from the eastern China, while the validation dataset included subjects from the northern and central China. No single SNP was validated in all three validation datasets suggests potential population stratification hence extrapolation of the result needs cautious implementation.

## 5 Conclusions

Based on a sequential investigation of single variant, gene-based and pathways enrichment analysis, we uncovered novel rare variants and genes that may contribute to the susceptibility of GI cancers. Further studies are warranted to look into the underlying mechanism of the association between these susceptibility markers and GI cancers.

## Data availability statement

The summary data of the detailed results of the study are included in the article/**Supplementary Material**. Further inquiries can be directed to the corresponding authors.

## Ethics statement

All patients provided an informed consent to participate in the study, and the study protocol was approved by the ethical committee of the Institutional Review Board of Fudan University Shanghai Cancer Center.

## Author contributions

The hypothesis of analyzing shared rare variants associated with the risk of GI cancer was conceived by RZ. JZ conducted quality control and bioinformatic analysis. Data collection and the bioinformatic analysis was conducted by XW. JL helped with the visualization of results. QW provided suitable guidance to the study design. MYW and YW helped with the collection of blood DNA samples. JC, JX, MLW and TW assisted with validation of significant SNPs in external datasets. All authors have read and approved the final manuscript.
