# Supplementary material for: Rare variants confer shared susceptibility to gastrointestinal tract cancer risk
Source: Front Oncol. 2023 Jul 6;13:1161639. doi: 10.3389/fonc.2023.1161639 (PMC10358854; doi:10.3389/fonc.2023.1161639)
Supplement: Supplementary file 1 [file DataSheet_1.docx]

**Supplementary Figure 1.** Types of rare variants annotated by ANNOVAR.

Abbreviations: IGR: Intergenic region mutation; Intron: Intronic mutation; RNA: Ribonucleic acid (RNA) mutation; 3’ UTR: 3’ Untranslated Region; 5’ UTR: 5’ Untranslated Region; in-frame/frame-shift ins/del: including in-frame insertion/deletion mutation, frame-shift insertion/deletion mutation.

**Supplementary Figure 2.** Manhattan plot for single variant association analysis in ESCC, GC and CRC.


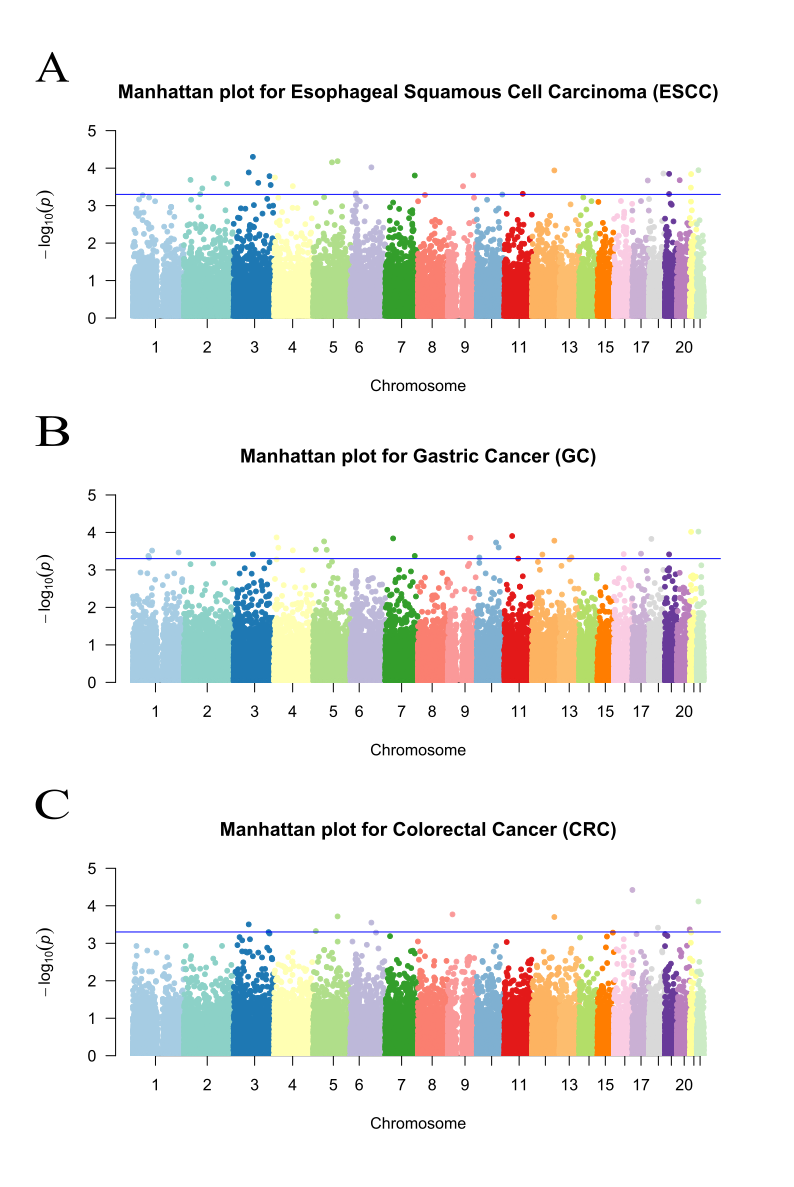


| A. Manhattan plot for Esophageal Squamous Cell Carcinoma (ESCC), 2,048 variants were significantly associated with the risk of ESCC. B. Manhattan plot for Gastric Cancer (GC), 2,182 variants were significantly associated with the risk of GC. C. Manhattan plot for Colorectal Cancer (CRC), 2,050 variants were significantly associated with the risk of CRC.  **Supplementary Figure 3.** Q-Q plot of logistic regression results analysis in ESCC, GC and CRC dataset. |
| --- |


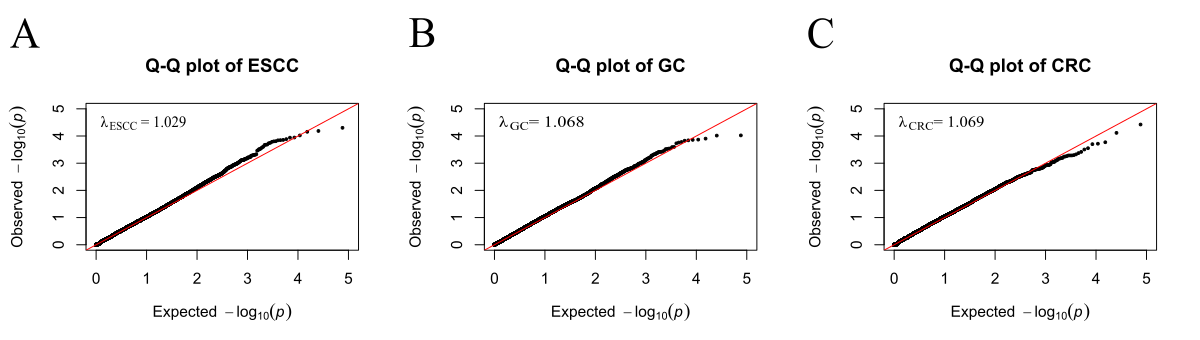


A. Q-Q plot of ESCC logistic model with λ_ESCC_ = 1.029. B. Q-Q plot of GC logistic model with λ_GC_ = 1.068. C. Q-Q plot of CRC logistic model with λ_CRC_ = 1.069. The Genomic inflation statistics (λ) showed modest deviations of observed *P*-value from expected *P*-value.
